# Dynamical and individualised approach of transcranial ultrasound neuromodulation effects in non-human primates

**DOI:** 10.1038/s41598-024-62562-6

**Published:** 2024-05-24

**Authors:** Cyril Atkinson-Clement, Mohammad Alkhawashki, James Ross, Marilyn Gatica, Chencheng Zhang, Jerome Sallet, Marcus Kaiser

**Affiliations:** 1 Precision Imaging, School of Medicine, University of Nottingham, Nottingham, UK; 2 Department of Neurosurgery, Ruijin Hospital, Shanghai Jiao Tong University School of Medicine, Shanghai, China; 3 Shanghai Research Center for Brain Science and Brain-Inspired Intelligence, Shanghai, China; 4 Department of Experimental Psychology, Wellcome Centre for Integrative Neuroimaging, University of Oxford, Oxford, UK; 5 Inserm, Stem Cell and Brain Research Institute U1208, Université Lyon 1, Bron, France; 6 School of Computing Science, Newcastle University, Newcastle upon Tyne, UK; 7 Rui Jin Hospital, Shanghai Jiao Tong University, Shanghai, China

**Keywords:** Focused ultrasound stimulation, Seed-based connectivity, Whole brain, Ultrasound, Animal model, Neurology, Computational neuroscience, Neuroscience

## Abstract

Low-frequency transcranial ultrasound stimulation (TUS) allows to alter brain functioning with a high spatial resolution and to reach deep targets. However, the time-course of TUS effects remains largely unknown. We applied TUS on three brain targets for three different monkeys: the anterior medial prefrontal cortex, the supplementary motor area and the perigenual anterior cingulate cortex. For each, one resting-state fMRI was acquired between 30 and 150 min after TUS as well as one without stimulation (control). We captured seed-based brain connectivity changes dynamically and on an individual basis. We also assessed between individuals and between targets homogeneity and brain features that predicted TUS changes. We found that TUS prompts heterogenous functional connectivity alterations yet retain certain consistent changes; we identified 6 time-courses of changes including transient and long duration alterations; with a notable degree of accuracy we found that brain alterations could partially be predicted. Altogether, our results highlight that TUS induces heterogeneous functional connectivity alterations. On a more technical point, we also emphasize the need to consider brain changes over-time rather than just observed during a snapshot; to consider inter-individual variability since changes could be highly different from one individual to another.

## Introduction

The recent advancement in low-intensity transcranial ultrasound stimulation (TUS) addresses several limitations of existing non-invasive brain stimulation modalities by enabling the alteration of brain function with an improved spatial precision and the capacity to target deep brain structures. Consequently, TUS holds the potential to represent a significant leap forward in the field of Neuroscience. However, due to its recent emergence, the mechanisms and effects of TUS remain largely unknow. Of interest to us are the effects of TUS beyond the target area, across the brain network and over-time.

TUS involves the application of mechanical sound pressure waves within the frequency range of 200 kHz to 10 MHz or beyond. This technique transiently modifies the function of brain cells without inducing tissue heating^1,2^. While its precise mechanism remains a subject of debate, several non-mutually exclusive hypotheses have been put forth. Firstly, TUS could activate voltage-gated sodium and calcium channels that respond to mechanical stimulation^3–5^. Secondly, it may induce localized depolarization by triggering expansions and contractions in brain cell membranes through microcavitation^6,7^. Thirdly it could impact on the coupling between glia cells and neurons^8^. Although TUS protocols should be applied with caution, experiments with monkeys have demonstrated that TUS induced neuromodulation without significant increased brain temperature at the target site^9^, without causing oedema or compromising the blood–brain barrier, without impacting on the integrating of the neural tissue^10,11^.

To date, a majority of studies examining the effects of TUS have focused on the immediate impact of TUS on brain function, the “*online*” effects. Additional investigations can elucidate how TUS may induce broader changes in brain function, encompassing the entire brain or specific sub-networks, over extended time periods. Existing evidence suggests that TUS could trigger transient effects ranging from several minutes to several hours, although many of the studies that focused on assessing the prolonged effects of TUS have primarily compared baseline conditions with post-TUS states, with limited consideration of the temporal dynamics of these effects.

In humans, TUS applied to regions linked to chronic pain improved mood significantly after 40 min^12^, while stimulation of the right inferior cortex induced positive mood effects lasting up to 30 min, along with changes in functional connectivity within the default mode network^13^. In monkeys, TUS applied to the anterior cingulate cortex led to altered cognitive performance during a counterfactual choice task more than 30 min after stimulation^14^, while TUS applied to cortical and deep cortical/subcortical regions has been shown to induce alterations in brain connectivity lasting over an hour^10,15^. Last, in vitro data showing that offline effects could last for up to 8–12 hours^16^.

**Figure 1:**
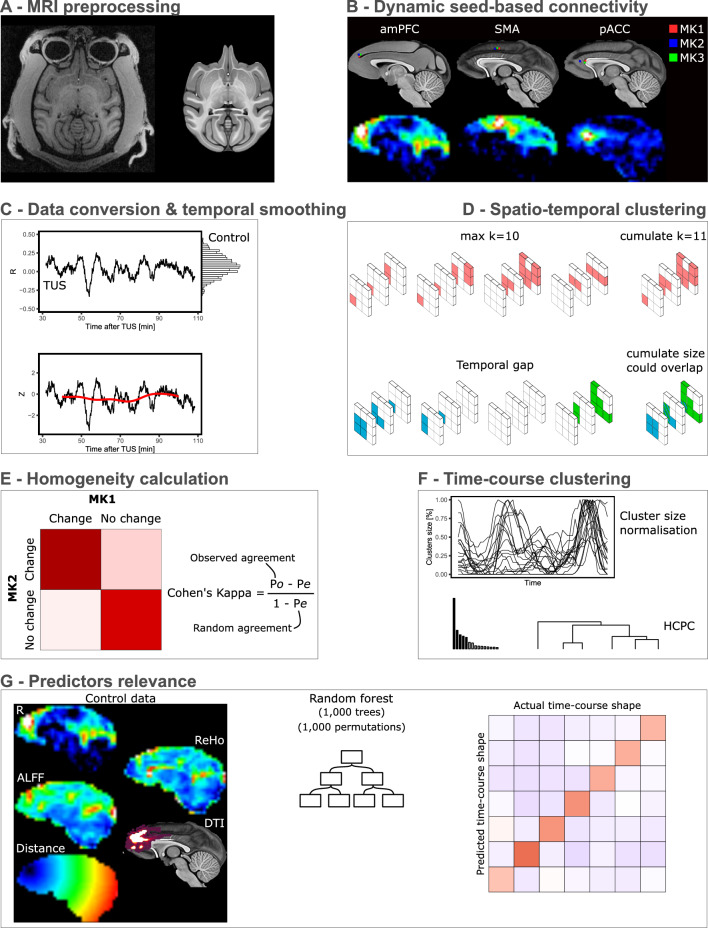
Overview of the analyses process. The panel (**A**) illustrates the rs-fMRI pre-processing while panel (**B**) shows the locations of the theoretical coordinates of each target for each individual monkey and an illustration of the seed-based functional connectivity for each of the target. The panel (**C**) shows the data conversion method from the raw R values to the Z-scored values smoothed using GAM and the panel (**D**) the spatio-temporal clustering method based on the DBSCAN algorithm. The panel (**E**) represents the homogeneity calculation based on the Cohen’s kappa and the panel (**F**) the time-course clustering approach to capture time effects. The panel (**G**) illustrates the data included in the random forest model to assess predictors importance as well as a contingency matrix between the predicted and actual time-courses observed in the PCA/HCPC model.

Our study endeavours to address this gap by investigating the dynamic propagation of TUS effects throughout the entire brain in three monkeys (details of each individual could be found in Table 1), targeting three distinct brain regions: the anterior medial prefrontal cortex (amPFC), the supplementary motor area (SMA) and the perigenual anterior cingulate cortex (pACC). This approach will facilitate the detection of TUS-induced effects over time, enabling the differentiation of changes that manifest or dissipate after TUS from those with longer-lasting implications. For each target and each monkey, (1) we computed the seed-based connectivity changes over-time using a sliding-window approach; (2) we assessed the inter-individual and inter-target homogeneity using Cohen’s kappa statistics; (3) we identified the temporal dynamics of all significant clusters; (4) and we assess if brain features obtained in the control condition could contribute to predict the outcomes we found using random forest coupled with permutations (see Fig. 1 for an overview of the analyses process). Our results highlight that (1) TUS alters brain functioning with a high inter-individual variability but with some consistent changes; (2) while most of the changes are transients, some could be of long duration (> 2 h); (3) with a significant accuracy, control brain features could contribute to predict how TUS alters brain functioning.

**Table 1:** Descriptive data of the 3 individuals and fMRI details.

	Age [years]	Delay between sedation/TUS and data collection [min]	Expired Isoflurane run1/run2/run3 [%]	Skull thickness/Distance skin to target [mm]
Target amPFC				
MK1	6.31	103/30	0.6/0.6/0.6	2.74/9.45
MK2	6.29	109/25	0.7/0.7/0.75	2.06/8.92
MK3	5.62	176/30	0.8/0.8/0.8	2.24/8.28
Target pACC				
MK1a	5.71	170/70	0.8/0.8/0.85	2.55/15.89
MK1b	5.86	94/55	0.8/0.8/0.85	2.55/15.89
MK2	5.97	105/40	0.8/0.8/0.8	2.12/15.79
MK3	5.33	172/30	0.8/0.75/0.7	2.91/18.12
Target SMA				
MK1	5.82	130/50	0.7/0.7/0.7	2.06/11.28
MK2	5.8	104/35	0.7/0.7/0.7	1.58/11.86
MK3	5.18	125/55	0.7/0.7/0.7	3.08/13.32

amPFC, Anterior medial prefrontal cortex; pACC, Perigenual anterior cingulate; SMA, Supplementary motor area.

## Results

### Seed-based functional connectivity changes after TUS

#### Anterior medial prefrontal cortex (amPFC) target

**Figure 2:**
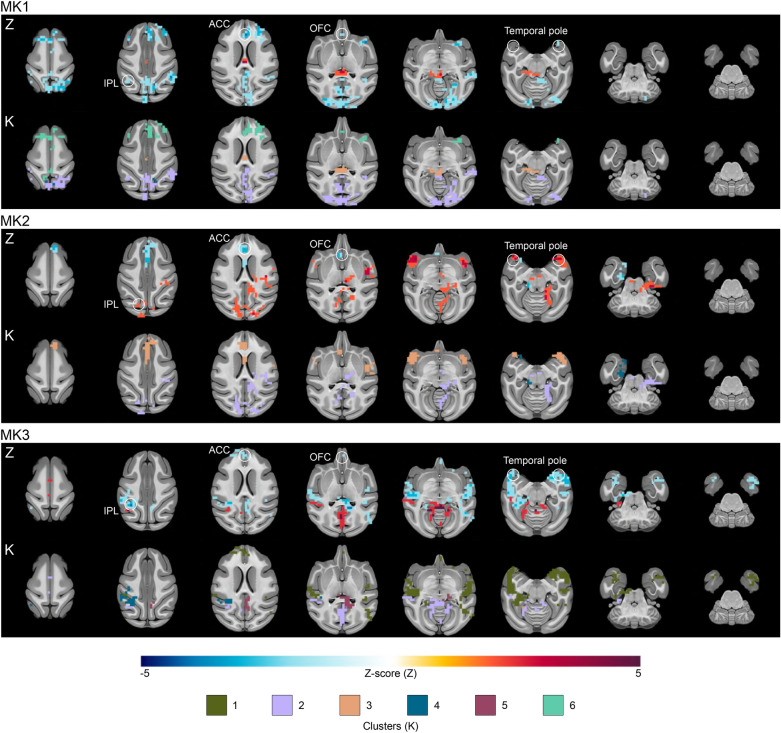
Anterior medial prefrontal cortex TUS effects on seed-based functional connectivity. The figure highlights the effects of amPFC-TUS for each monkey by showing the significant voxels found over-time (lines Z) and the clusters on the basis of the PCA/HCPC model to identify the shape of the time-courses (lines K). ACC: anterior cingulate cortex; IPL: inferior parietal lobule; OFC: orbitofrontal cortex.

For the amPFC target (Fig. 2 and Table S1), prominent clusters (k ≥ 100 voxels) were observed in the right inferior parietal lobule (IPL), the medial dorsolateral prefrontal cortex (DLPFC), the right parahippocampus, the left inferior temporal cortex (ITC), the right superior temporal cortex (STC), and the left primary visual cortex (V2). Notably, consistent patterns of reduced connectivity emerged between the seed region and the anterior cingulate cortex (ACC) and the medial orbitofrontal cortex (OFC). Conversely, diverse connectivity changes were observed across the left IPL and the bilateral temporal pole.

#### Supplementary motor area (SMA) target

**Figure 3:**
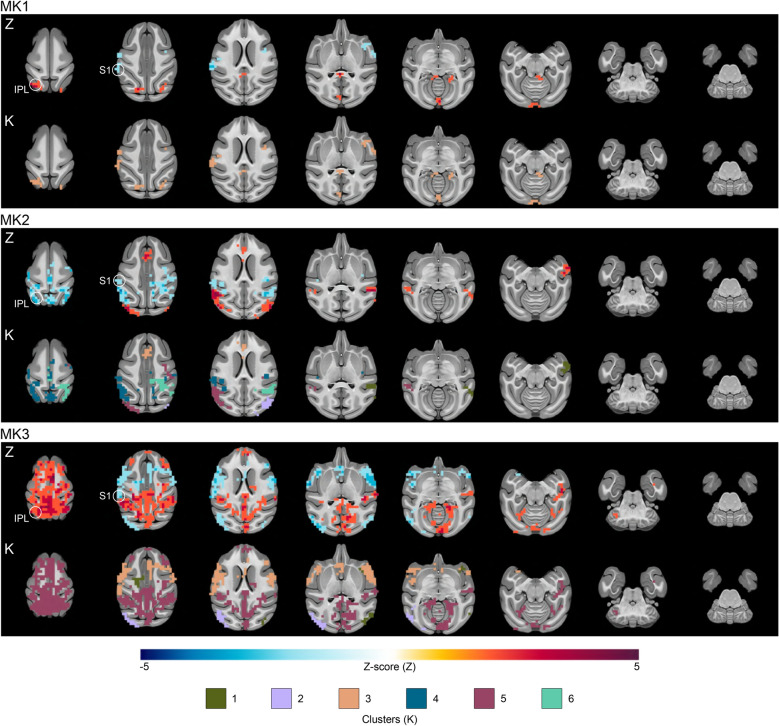
Supplementary motor area TUS effects on seed-based functional connectivity. The figure highlights the effects of SMA TUS for each monkey by showing the significant voxels found over-time (lines Z) and the clusters on the basis of the PCA/HCPC model to identify the shape of the time-courses (lines K). IPL: inferior parietal lobule; S1: primary sensory cortex.

For the SMA target (Fig. 3 and Table S1), the most substantial clusters (k ≥ 100 voxels) were localized in the right IPL, the right premotor cortex, the left primary somatosensory cortex (S1), the right STC, the left extrastriate visual cortex (V4), and the left primary visual cortex (V1). A consistent reduction in connectivity was observed between the seed region and the left S1. Further differences were noted in the connectivity between the left IPL and SMA, with differing patterns among the monkeys.

#### Perigenual anterior cingulate cortex (pACC) target

**Figure 4:**
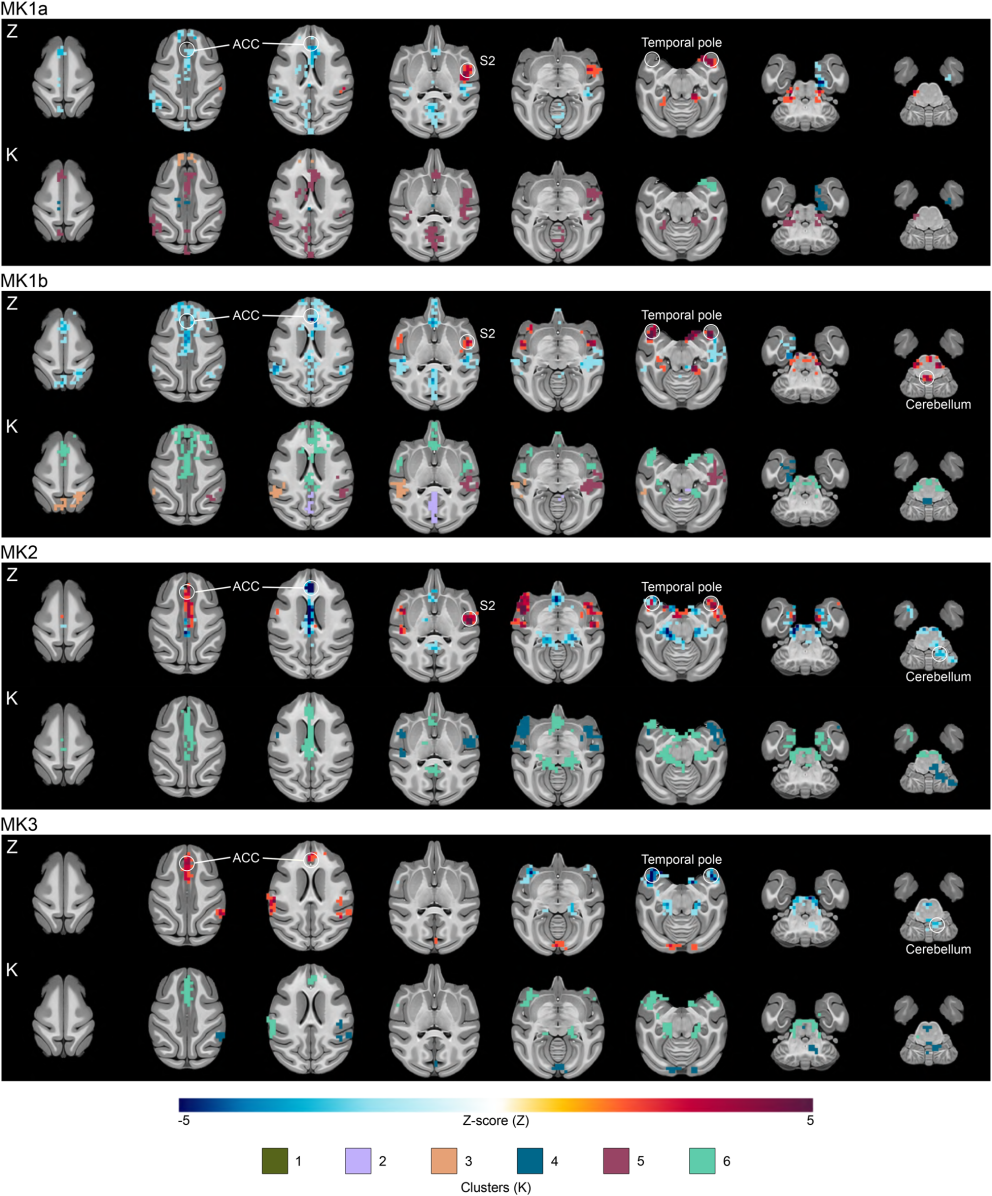
Perigenual anterior cingulate cortex TUS effects on seed-based functional connectivity. The figure highlights the effects of pACC TUS for each monkey by showing the significant voxels found over-time (lines Z) and the clusters on the basis of the PCA/HCPC model to identify the shape of the time-courses (lines K). ACC: anterior cingulate cortex.

For the pACC target (Fig. 4 and Table S1), the largest clusters (k ≥ 100 voxels) were found in the pACC, the left hippocampus, the right amygdala, the right auditory cortex, the medial V1, and the right secondary somatosensory cortex (S2). Changes were observed in the pACC connectivity across all four TUS sessions (decreased twice, increased once, and one mixed result). There were also variations bilaterally in the connectivity of the temporal pole with increased connectivity twice, decreased connectivity once, and a mixed result with both an increased and a decreased connectivity.

#### Homogeneity of TUS effects on functional connectivity

**Figure 5:**
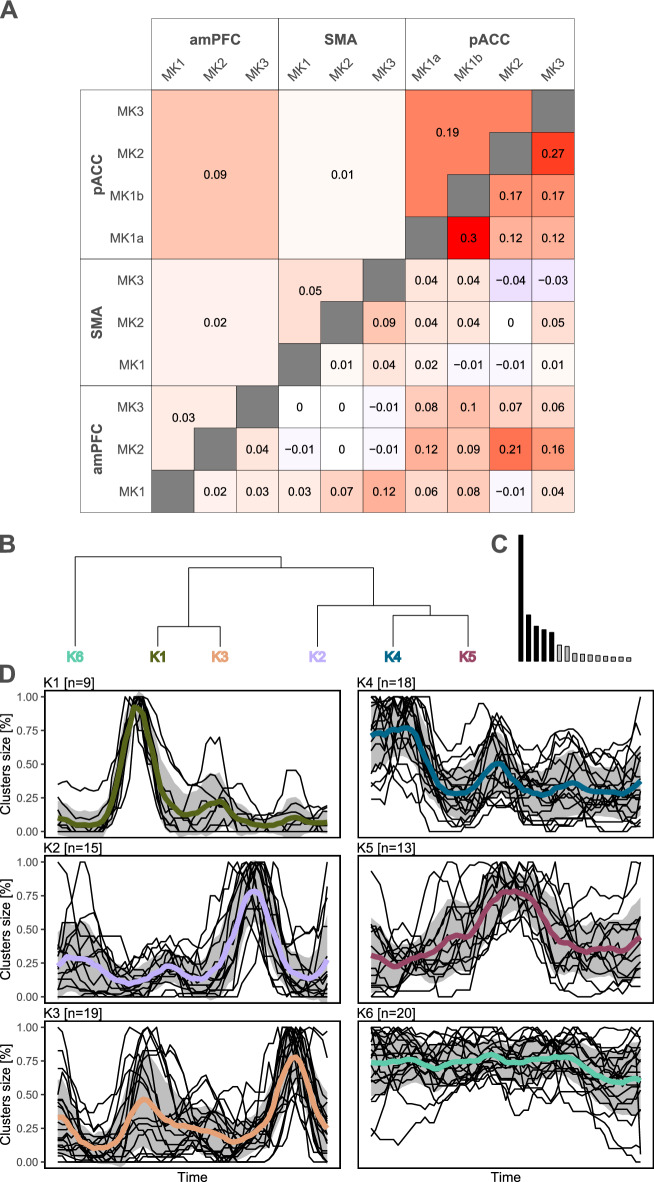
Homogeneity and time-courses of functional connectivity changes. The panel (**A**) shows the homogeneity of functional connectivity changes observed between monkeys and targets. Values close to 1 correspond to a high homogeneity, values close to 0 corresponds to a high heterogeneity, while values close to − 1 correspond to opposite results. The panels (**B**) and (**C**) show the results of the PCA/HCPC clustering. The panel (**D**) shows the 6 time-courses observed for each cluster (black lines) and the mean of each cluster (bold lines). amPFC, anterior medial prefrontal cortex; pACC, perigenual anterior cingulate; SMA, supplementary motor area.

To gauge the homogeneity of TUS effects among and between different targets, Cohen's Kappa score was employed (Fig. 5A). The Kappa scores for the same monkey versus different monkeys were not significantly different (same monkey: 0.059 ± 0.099; different monkeys: 0.063 ± 0.066; F_(1;43)_ = 0.022; *p* = 0.883), while higher Kappa scores were evident when the same target was stimulated, as opposed to different targets (same target: 0.116 ± 0.097; different targets: 0.043 ± 0.056; F_(1;43)_ = 9.925; *p* = 0.003). In addition, when we compared the Kappa obtained for the same target, the homogeneity was notably greater from stimulating the pACC target (0.191 ± 0.079) compared to the SMA or amPFC targets (0.051 ± 0.04 and 0.031 ± 0.011; F_(2;9)_ = 8.784; *p* = 0.008 respectively).

### Time courses of TUS effects on functional connectivity

To characterize the temporal dynamics of TUS effects on functional connectivity, we focused on the clusters identified as significant. We normalized the values of these clusters based on their own size percentage. PCA was employed to reduce data complexity, retaining the first five components (84.803% of the total variance). Subsequently, HCPC led to the identification of six noteworthy time-clusters (Fig. 5B–D). Four of these exhibited fluctuating effects characterized by cluster size variations (K1: short-term; K5: medium-term; K2: long-term; K3: both short and long-term). In contrast, two time-clusters displayed more linear trajectories (K4: high significance in the short-term with a gradual reduction in size; K6: stable from the beginning to the end of the time window).

Delving further, we observed spatial consistencies in these time-clusters (Figs. 2, 3 and 4). Specifically, K1 was predominantly located in temporal regions. K2 was exclusively situated in posterior brain regions (parietal, occipital, and posterior cingulate cortices), regardless of the target region. K3 frequently emerged within the TUS target region or in its vicinity, as well as in temporal regions following. K4 was frequently located in limbic areas, including the hippocampus and the insula, and in cerebellar regions. K5, a relatively substantial cluster, was directly linked to the target region. Lastly, K6 was predominantly linked to the target region, notably observed after amPFC-TUS and pACC-TUS. Furthermore, K6 displayed strong connections with deep brain areas, including the pons, medulla, hippocampus, and amygdala.

### Relevance of TUS effects predictors

**Figure 6:**
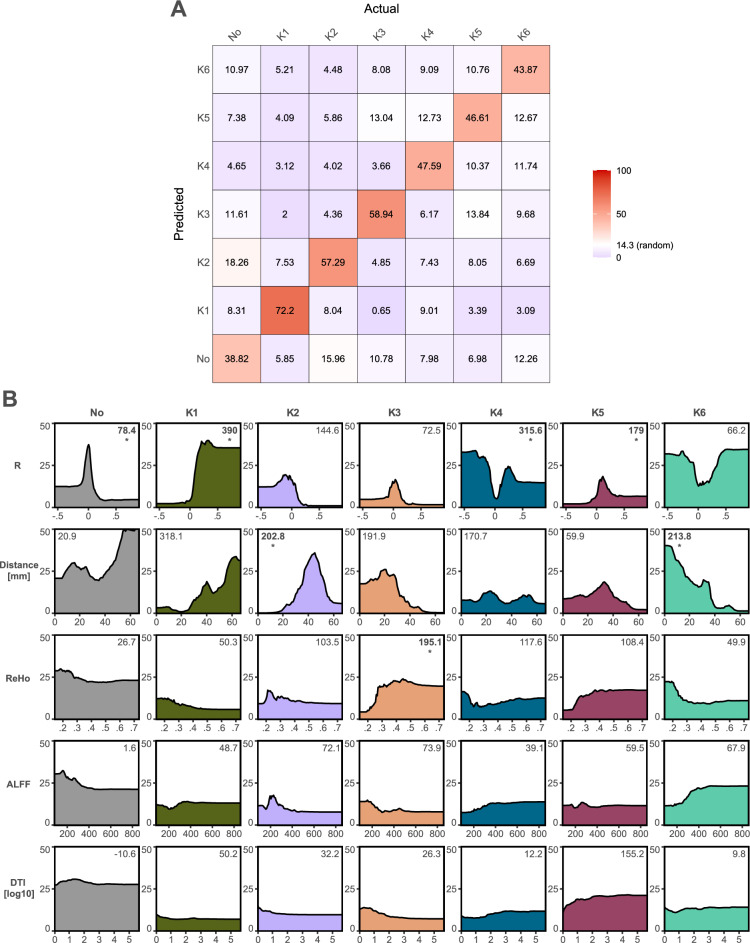
Relevance of brain features to predict changes following TUS. The panel (**A**) shows the contingency matrix between the actual time-courses of changes following TUS and the one predicted by the random forest model, regardless of the target and the individual. The panel (**B**) highlights the marginal predictions for each predictor on each time-course (as probability to happen). The more relevant feature was highlight with MDA values in bold and follow by a “*”.

Drawing from the six identified time-course patterns and an additional seventh group representing no TUS effects, we constructed a random forest model employing permutation-based techniques. Remarkably, the overall model exhibited a notable accuracy of 39.8% (random accuracy threshold: 14.28%; *p* < 0.0001; Fig. 6A). Among the groups, accuracy ranked as follows: K1 (72.2%), K3 (58.9%), K2 (57.3%), K4 (47.6%), K5 (46.6%), K6 (43.9%), and the absence of TUS effects (38.8%). Three predictors emerged as significantly relevant overall: seed-based connectivity (MDA = 145.65, *p* = 0.0009), Euclidean distance from the target centre (MDA = 52.88, *p* = 0.0009), and ReHo (MDA = 44.08, *p* = 0.0009). In contrast, ALFF and structural connectivity were found as not relevant (MDA < 11.13, *p* > 0.052).

Examining individual predictors, seed-based connectivity emerged as the most significant predictor for K1, K4, K5, and the group with no TUS effects. Meanwhile, Euclidean distance proved most influential for K2 and K6, while ReHo exhibited greater significance for K3 (Fig. 6B).

## Discussion

While the causal impact of TUS at the cellular and circuit levels have been extensively investigated^8,10,15–18^, no study have investigated the dynamical effects of transcranial ultrasound stimulation (TUS). We investigated this issue with an individual-based approach. By using TUS on three distinct brain targets, we have unveiled significant insights: (1) TUS prompts heterogenous functional connectivity alterations that exhibit inter-individual variance, yet retain certain consistent changes across individual monkeys; (2) we identified several time-courses of these changes whose with some are being transient while others were of longer duration (> 2 h); (3) remarkably, our findings demonstrate the ability to predict changes with a notable degree of accuracy using pre-existing neural characteristics for which 3 were significantly relevant: seed-based functional connectivity, Euclidean distance from the target, and ReHo.

### TUS effects are mostly of high heterogeneity

Although our results demonstrated Cohen's Kappa values significantly surpassing randomness, indicating a certain level of consistency, the overarching observation is that inter-individual heterogeneity remains pronounced. Several factors could explain the inter-individual variability in functional connectivity (Table 1). Skull thickness variability could impact on the efficacy of the stimulation. This factor was not taken into account in the original studies those data were initially collected for. Later blackbone MRI scans^19^ revealed some differences in the thickness of the skull over the stimulations sites in the 3 animals studied. While depth of anaesthesia was similar across the 3 animals, variability in delay between stimulation and data collection is a second factor that could explain some of variance observed.

Overall this underscores the imperative for personalised interventions^20^ and the use of computational modelling in predicting optimal targets and parameters for individual subjects^21^.

Nevertheless, we identified numerous changes that are shared across monkeys, thereby reinforcing the significance of our findings. TUS targeted at amPFC primarily perturbed brain dynamics in anterior cerebral regions (e.g., ACC and OFC). Stimulation of the SMA influenced the somatosensory-motor pathway (e.g., S1 and IPL). Conversely, TUS directed at the pACC predominantly yielded localized and transient effects (e.g., pACC's self-connectivity and the temporal pole). These alterations frequently manifested as reductions in functional connectivity, implying that TUS disrupted the coupling between the target regions and the corresponding networks. Even if initially unexpected, similar results were already observed with other neuromodulation approached (e.g., for M1 neuromodulation: increased^22^, decrease^23^ or variables^24^ changes of local connectivity). It should be noted that these effects may be influenced by the specific ultrasound neuromodulation parameters employed in our study. Although there is an emerging body of evidence linking stimulation parameters to effects at the targeted site^25^, the broader impact of these parameters on brain networks beyond the target area remains enigmatic.

### TUS effects could be of long duration

Our analysis also revealed that TUS induces different changes over-time. While certain effects exhibited transient characteristics (designated as K1, K2, K3, and K5), others displayed a progressive reduction (as exemplified by K4) or persisted as stable, long-term alterations (illustrated by K6).

On the one hand, the observed duration of effects aligns with prior findings indicating shifts in brain connectivity spanning from 30 min^13,14^ to 60 min^10,15^. For instance, investigations in healthy individuals have demonstrated that TUS applied to the sensorimotor network can trigger sustained network enhancement for up to a week following stimulation^26^. On the other hand, it also suggests that studies which used a medium-term snapshot approach to identify brain changes following TUS could both conclude about transient changes or miss some effects. Similar outcomes were already reported following brain stimulation, suggesting that some changes could be restricted to the stimulation time, be transient, or even start sometimes after the end of the stimulation^24,27,28^.

In addition, this time pattern is more complex than what would be expected from an initial rise of an effect with a following decrease over-time. It highlights that different brain regions, possibly due to their internal organisation, their connectivity with other brain regions, and their current state, show distinct time courses of ultrasound effects.

### TUS effects could be anticipated

Finally, we completed our analysis by using a random forest model to ascertain the significance of pre-TUS brain features in predicting alterations in brain function resulting from TUS. While the accuracy was suboptimal to use for prediction, it still surpassed random chance and thereby allowed us to delineate three specific data categories that are relevant: seed-based functional connectivity, Euclidean distance from the target, and ReHo. Interestingly, structural connectivity strength was not a relevant predictor. This could be explained by the fact that larger fibre tracts are often due to connecting larger brain regions without necessarily facilitating the spreading of neuromodulation effects throughout the network^29–31^. While this approach represents a novel application within the field of focused ultrasound neuromodulation, it is important to note that similar methodologies have been previously employed. For instance, this technique has been utilised to forecast potential responses among individuals with schizophrenia to various treatments based on their rs-fMRI profiles^30^. Additionally, it has been employed to predict alterations in mood and cognitive functioning following transcranial direct current stimulation in patients diagnosed with major depression, utilizing rs-EEG data^31^. Furthermore, this methodology has been employed to predict the severity of Parkinson's disease by analysing the rs-fMRI ALFF metric^29^. As further investigations unfold, it will be imperative to validate the predictive efficacy of this particular pattern. If validated, such an approach could potentially pave the way for a more personalized and tailored implementation of focused ultrasound neuromodulation.

## Conclusion

In conclusion, our study highlights that TUS effects on functional connectivity vary over-time: measuring the effect after say half an hour will give a different result from measuring after one hour as some effects only become visible a long time after stimulation. This will be a challenge for comparing studies in the field. We therefore strongly suggest observing the time course of functional connectivity changes over-time rather than just observing a snapshot at one time point. Our study also highlights the heterogenous time courses for different brain regions with the influence of connectivity, state, target, and parameters as future areas of research. Finally, we show that effects are stable in some regions, being present for the whole duration of the rs-fMRI, indicating that long-term effects over several hours or days in non-human primates might be feasible.

## Methods

### Subject details

Data from healthy adults’ male rhesus macaques (*Macaca mulatta*) were used for this study. Three animals participated to this study which involved 3 TUS targets: amPFC, SMA, pACC. Finally, scans were also collected without prior transcranial stimulation (average age at time of scan: 6.35 years). Details of each individual could be found in Table 1.

All procedures were conducted under project and personal Animals Scientific Procedures e-Licensing (ASPel) licences evaluated and approved by the United Kingdom (UK) Home Office in accordance with the UK The Animals (Scientific Procedures) Act 1986, the European Union (EU Directive 2010/63/EU) and the ARRIVE guidelines.

### Ultrasound stimulation

A single element ultrasound transducer (H115-MR, diameter 64 mm, Sonic Concept) with 51.74 mm focal depth was used with region-specific coupling cones filled with degassed water and sealed with a latex membrane. The pulse repetition frequency of ultrasonic wave was set at 250 kHz with 30 ms bursts of ultrasound generated every 100 ms, controlled through a digital function generator (Handyscope HS5, TiePie engineering). The stimulation lasted for 40 s. Each of the areas targeted lie close to the midline. Therefore, we applied a single train over the midline stimulating the target region in both hemispheres simultaneously. Based on numerical simulations (described in^10,15^), the maximum peak pressure and I_sspa_ at the acoustic focus point were estimated to be 1.01 MPa and 31.7W/cm^2^ for the amPFC (I_spta_: 9.5W/cm^2^), 0.88 MPa and 24.1W/cm^2^ for the SMA (I_spta_: 7.2W/cm^2^), and 0.78 MPa and 18.8W/cm^2^ for the pACC (I_spta_: 5.63W/cm^2^). The maximum increased of temperature was estimated to be + 2.9 °C in the skull, + 1 °C in the dura, and + 0.5 °C in the brain^10^. No adverse effect was observed nor any tissue alteration^10^.

In order to direct TUS to the target region, we guided the stimulation using a frameless stereotaxic neuronavigation system (Rogue Research) set up for each animal individually by registering the T1-weighted image to the animal’s head. Positions of both the ultrasound transducer and the head of the animal were tracked continuously with infrared reflectors to inform online and accurate positioning of the transducer over the targeted brain region (amPFC [x = − 0.7, y = 24, z = 11]; SMA [x = 0.1, y = 2, z = 19]); pACC [x = 0, y = 15, z = 6]). The ultrasound transducer/coupling cone montage was placed directly onto previously shaved skin prepared with conductive gel (SignaGel Electrode) to ensure ultrasonic coupling between the transducer and the animal's scalp. A minimum of 10 days elapsed between two TUS session. In the non-stimulation condition (control), all procedures, with the exception of actual TUS, matched the TUS sessions. Each stimulation occurred in a specific session. The first animal (MK1) was involved in four TUS sessions (one in the PFC, one in the SMA and two in the pACC), while the second (MK2) and third (MK3) animals were involved in three sessions (one per target).

### Neuroimaging data acquisition

For each monkey and each target, we acquired one MRI. First, monkeys were anaesthetised using inhalational isoflurane gas, based on an already used protocol which preserve whole-brain functional connectivity^32–34^. Monkeys also received intramuscular injection of ketamine (10 mg/kg), xylazine (0.125–0.25 mg/kg), midazolam (0.1 mg/kg) and atropine (0.05 mg/kg), and intravenously of meloxicam (0.2 mg/kg) and ranitidine (0.05 mg/kg). Following the TUS application, the animals were placed in a sphinx position in a 3 T MRI scanner. Scanning commenced approximately 2 h following anaesthesia to avoid the clinical peak of ketamine. Physiological parameters were monitored to verify depth of anaesthesia, heart rate, blood pressure, clinical verifications for muscle relaxation. Intermittent positive pressure ventilation was maintained to ensure a constant respiration rate. Respiration rate, inspired and expired CO_2_ and isoflurane concentration were monitored and recorded using VitalMonitor software. Core temperature and SpO_2_ were also constantly monitored throughout the scan.

For each session, three fMRI data were collected as follows: 36 axial slices; in-plane resolution: 2 × 2 mm; slice thickness: 2 mm; no slice gap; TR: 2000 ms; TE: 19 ms; 800 volumes per run; approximately 26 min per run).

In addition, a structural scan was acquired for each monkey using a T1 weighted magnetization-prepared rapid-acquisition gradient echo sequence (voxel resolution: 0.5 × 0.5 × 0.5 mm), as well as a DWI (voxel resolution: 1 × 1 × 1 mm) and a Black bone (voxel resolution: 0.5 × 0.5 × 0.5 mm).

### Neuroimaging data pre-processing and metrics extraction

The preprocessing of resting-state functional magnetic resonance imaging (rs-fMRI) data was conducted using the AFNI software^35,36^, following standard preprocessing procedures. Initially, the T1-weighted image was aligned with the rs-fMRI data. Subsequently, the T1 image underwent preprocessing steps involving skull removal, tissue segmentation, and alignment with a template space. The rs-fMRI data underwent preprocessing steps including slice timing correction, de-spiking, and motion correction within the native space, followed by alignment to the mean image. The partially preprocessed rs-fMRI data were then aligned to a template space using the conversion matrix derived from the T1 preprocessing. Furthermore, detrending was performed on the rs-fMRI data using motion as a nuisance variable, followed by spatial smoothing (3 mm). At this juncture, a dynamic map was generated through a sliding window approach (window size of 100 volumes moving by 1 volume) to capture seed-based functional connectivity. This procedure encompassed all monkeys and regions of interest (ROIs), employing a 3 mm sphere centred on the theoretical coordinates of each target for each individual monkey (Fig. 1A, B).

### Statistics and reproducibility

All the statistical analyses were achieved using R^37^ and are illustrated in Fig. 1.

#### Seed-based connectivity changes over-time

We evaluated changes in seed-based connectivity over-time on an individual basis for each monkey and TUS target.

First, for each voxel, we extracted connectivity values from both the TUS condition and the corresponding control rs-fMRI dataset (pertaining to the same monkey and seed ROI). These values were then transformed into Z-scores (see Fig. 1 panel C):

$$Z=\frac{x- \mu }{\sigma }$$

where $x$ is the individual value (following TUS), $\mu$ is the mean of the control condition and $\sigma$ the standard deviation of the control condition. Then, each $Z$ value was associated with its corresponding time value. For example, when constructing connectivity maps based on volumes acquired within a time interval (e.g., between 30 and 32 min after TUS), a representative value (e.g., 31) was selected. To smooth the Z-score values over-time, we computed for each voxel a generalised additive model (GAM^38^) which automatically selected the smoothing parameters with a restricted maximum likelihood approach. We then reduced temporal resolution by only selecting predicted values beginning from 10 min after the onset of rs-fMRI and ending 10 min before its conclusion, thus excluding border values for enhanced prediction quality, and by selecting only one value per minute. For example, for a rs-fMRI which started 30 min after TUS and finished 110 min after (1800 TR), we selected 1 value per minutes from 40 to 100 min after TUS (61 values).

To identify significant clusters, we generated two binarized 4D images in order to only keep voxels with an estimated Z-score higher than + 2 or lower than − 2. We then used a density-based spatial clustering (DBSCAN^39^; Fig. 1D) on the 4D coordinates of all significant voxels (x, y, z, time) with a maximum Euclidean distance of 1.5units (which corresponds to the share of at least one face) between two significant voxels of the same clusters. Then, only clusters for which the size reached 20 voxels at least once for a specific time point were considered as significant.

#### TUS effects homogeneity assessment

In order to assess the homogeneity of the TUS effects we identified, we used the Cohen’s kappa statistic^40^ (Fig. 1E):

$$K=\frac{Po-Pe}{1-Pe}$$

where $Po$ is the observed agreement and $Pe$ is the hypothetical probability of chance agreement. Specifically, for each monkey and each TUS target, we generated a data frame with all the voxels coordinates (n = 11,500) and if they were found to be significantly changed (1) or not (0). Then, we compared each data frame with the other to extract the Cohen’s kappa. A value between − 1 and 0 refers to a disagreement between the two compared conditions, while a value between 0 and 1 refers to an agreement. Also, the more the value will be close to − 1/1 more the disagreement/agreement will be strong, while a value close to 0 refers to a lack of agreement. Furthermore, we computed mean values for each condition to evaluate intra- and inter-target relevance. This evaluation aimed to discern whether targeting the same site across multiple individuals would yield a more consistent homogeneity of TUS effects compared to targeting distinct sites across various individuals (random effect).

#### TUS effects dynamic clustering

To capture the over-time dynamics of TUS effects, we used a clustering approach based on the scaled cluster size (i.e., the size of each significant cluster was scaled to the percentage of their maximal size [between 0 and 1] at each time point). We then applied a principal component analysis (PCA) followed by a hierarchical clustering on principal components (HCPC^41^; Fig. 1F). The objective of the PCA was to reduce the data complexity by selecting the first 5 principal components (default parameter). Then the HCPC was applied to identify the optimal number of similar time dynamic (i.e., clusters) by using the Ward’s criterion (i.e., consisting of selecting the number of clusters which will allow to obtain the lower within-cluster variance [so-called inertia] and the higher between-cluster variance).

#### TUS effects predictors

Lastly, we used MRI controls data to predict the TUS effects across time (i.e., the cluster of each significant voxel). To achieve this, we extracted the seed-based functional and structural connectivity (using the DTI sequences, and normalised with log10), the Euclidean distance from the target, as well as the Amplitude of Low-Frequency Fluctuations (ALFF; corresponding to a measure of spontaneous fluctuations which could be interpreted as spontaneous neural activity^42,43^) and the Regional Homogeneity (ReHo; corresponding to the local functional connectivity between a voxel and its nearest neighbouring which could be interpreted as local synchronisation^44^) metrics. All these values were inserted into a random forest model based on permutations^45^ (Fig. 1G) allowing us to compare the relevance of each metric in comparison to a null distribution (1000 trees per random forest and 1000 permutations to build the null distribution). The relevance of each metric was measure by the mean decrease accuracy (MDA) after normalisation obtained as follows:

$$MDA=\frac{\mu (DA)}{\sigma (DA)}$$

where $\mu (DA)$ represents the mean decrease accuracy of trees and $\sigma (DA)$ represents the standard deviation of the decrease accuracy of trees. The obtained values could not be interpreted as an error rate or error counts, but more as a statistic test for which a high value refers to a significant utility of the predictor while a low/negative value means that the predictor is useless.

Then, we computed the marginal effects^46^ for each predictor and each modality of the variable to predict (i.e., each cluster) to determine which predictor’s values led to the maximal probability to induce a specific TUS effect.

### Supplementary Information

Supplementary Figure S1.

Supplementary Figure Legend.

Supplementary Table S1.

## Data Availability

The dataset and codes used in the current study are available from the corresponding author upon reasonable request.
