# Supplementary figures and images for: Dynamical and individualised approach of transcranial ultrasound neuromodulation effects in non-human primates
Source: Sci Rep. 2024 May 24;14:11916. doi: 10.1038/s41598-024-62562-6 (PMC11126417; doi:10.1038/s41598-024-62562-6)

amPFC

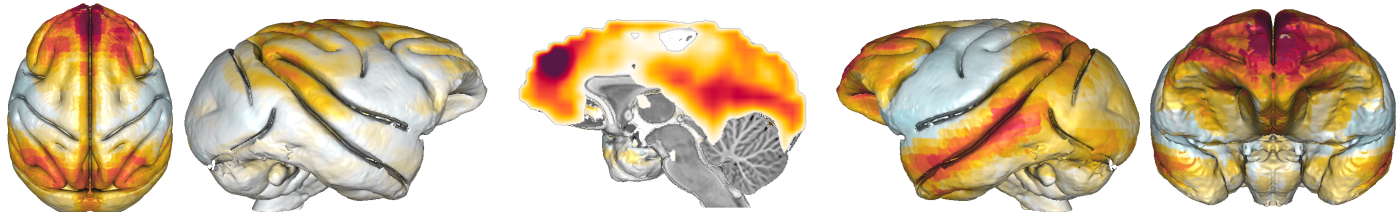

pACC

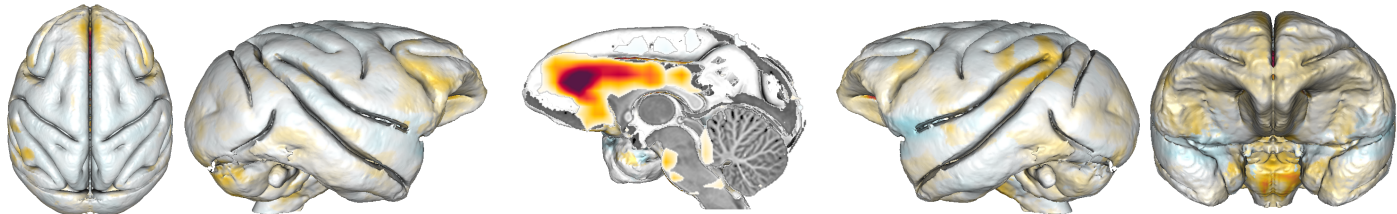

SMA

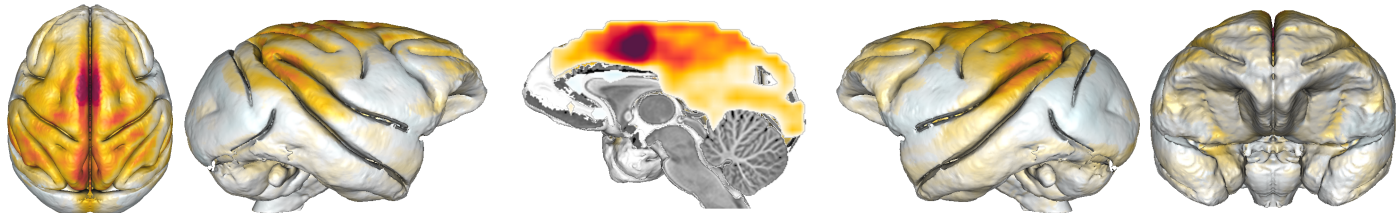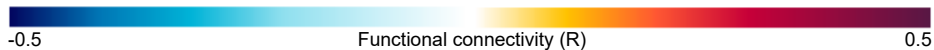

Supplement: Supplementary file 1 — Supplementary Figure S1. [file 41598_2024_62562_MOESM1_ESM.pdf]
