# Supplementary material for: Dynamical and individualised approach of transcranial ultrasound neuromodulation effects in non-human primates
Source: Sci Rep. 2024 May 24;14:11916. doi: 10.1038/s41598-024-62562-6 (PMC11126417; doi:10.1038/s41598-024-62562-6)
Supplement: Supplementary file 2 — Supplementary Figure Legend. [file 41598_2024_62562_MOESM2_ESM.docx]

**Figure S1. Seed-based functional connectivity during the control condition.**

The figure highlights the resting-state seed-based functional connectivity obtained during the control condition for the three animals (average) and the three targets.

amPFC: anterior medial prefrontal cortex; pACC: perigenual anterior cingulate; SMA: supplementary motor area.
