# Supplementary material for: Dynamical and individualised approach of transcranial ultrasound neuromodulation effects in non-human primates
Source: Sci Rep. 2024 May 24;14:11916. doi: 10.1038/s41598-024-62562-6 (PMC11126417; doi:10.1038/s41598-024-62562-6)
Supplement: Supplementary file 3 — Supplementary Table S1. [file 41598_2024_62562_MOESM3_ESM.docx]

**Table S1. Details of the significant clusters for each monkey and each target.**

| **Peak** | | | | | **Cluster** | | | |  |
| --- | --- | --- | --- | --- | --- | --- | --- | --- | --- |
| **x** | **y** | **z** | **t** | **Z-score** | **Time range** | **Size (max)** | **Size (cumul)** | **Time course pattern** | **Label** |
| **MK1 (PFC: 40’ – 100’)** | | | | | | | | | |
| 1 | 38 | 32 | 83 | -5.45 | 40 – 100 | 119 | 273 | K6 | Orbitofrontal cortex (medial) |
| 1 | 2 | 16 | 59 | 4.77 | 40 – 100 | 30 | 61 | K3 | Epithalamus (right) |
| 19 | 0 | 28 | 78 | -4.06 | 40 – 100 | 250 | 465 | K2 | Inferior parietal lobule (right) |
| -19 | -2 | 32 | 84 | -3.8 | 40 – 100 | 20 | 37 | K2 | Inferior parietal lobule (left) |
| -1 | -18 | 20 | 100 | -3.77 | 92 – 100 | 27 | 28 | K2 | Visual area V2 (left) |
| 25 | 2 | 14 | 83 | -3.45 | 60 – 91 | 23 | 29 | K2 | Inferior temporal cortex (right) |
| 3 | -8 | 34 | 44 | -3.07 | 40 – 59 | 28 | 37 | K4 | Superior parietal lobule (right) |
| **MK2 (PFC: 35’ – 95’)** | | | | | | | | | |
| -11 | 10 | 6 | 62 | -4.76 | 35 – 95 | 25 | 53 | K4 | Hippocampus (left) |
| -19 | 26 | 14 | 55 | 4.22 | 35 – 95 | 42 | 47 | K3 | Caudal orbitofrontal cortex (left) |
| 7 | -18 | 26 | 79 | 3.89 | 73 – 88 | 21 | 22 | K2 | Visual area V2 (right) |
| 15 | 4 | 4 | 72 | 3.87 | 56 – 95 | 163 | 248 | K2 | Parahippocampus (right) |
| 19 | 26 | 10 | 59 | 3.77 | 46 – 76 | 57 | 71 | K3 | Temporal pole (right) |
| -1 | 32 | 22 | 51 | -3.69 | 35 – 74 | 49 | 93 | K3 | Anterior cingulate cortex (left) |
| 13 | 26 | 10 | 87 | 3.25 | 74 – 95 | 23 | 28 | K3 | Caudal orbitofrontal cortex (right) |
| 19 | 26 | 10 | 35 | 3.23 | 35 – 44 | 29 | 29 | K3 | Temporal pole (right) |
| -3 | -18 | 26 | 79 | 3.23 | 72 – 89 | 42 | 55 | K2 | Visual area V1 (left) |
| -1 | 26 | 28 | 87 | -3.07 | 82 – 95 | 54 | 62 | K3 | Anterior cingulate cortex (left) |
| **MK3 (PFC: 40’ – 100’)** | | | | | | | | | |
| 23 | 22 | 8 | 57 | -4.48 | 40 – 100 | 124 | 214 | K1 | Rostral superior temporal cortex (right) |
| -13 | 0 | 28 | 48 | -4.26 | 40 – 100 | 45 | 71 | K4 | Inferior parietal lobule (left) |
| 21 | -6 | 12 | 55 | -4.12 | 50 – 70 | 36 | 48 | K1 | Visual area V3 (right) |
| -23 | 8 | 12 | 62 | -4.07 | 40 – 100 | 122 | 229 | K1 | Rostral superior temporal cortex (left) |
| -13 | -4 | 8 | 87 | 4 | 40 – 100 | 92 | 131 | K2 | Intermediate cerebellar cortex (left) |
| -15 | -4 | 26 | 82 | 3.5 | 57 – 100 | 32 | 36 | K2 | Inferior parietal lobule (left) |
| -29 | 4 | 16 | 50 | 3.45 | 40 – 56 | 20 | 25 | K2 | Inferior temporal cortex (left) |
| -1 | 10 | 36 | 86 | 3.38 | 81 – 95 | 20 | 22 | K2 | Primary motor cortex (left) |
| 3 | 38 | 20 | 59 | -3.31 | 48 – 79 | 20 | 37 | K1 | Lateral orbitofrontal cortex (right) |
| **MK1 (SMA: 60’ – 120’)** | | | | | | | | | |
| -1 | 2 | 18 | 111 | 4.71 | 98 – 119 | 33 | 41 | K3 | Epithalamus (left) |
| -13 | -8 | 34 | 110 | 4.02 | 75 – 119 | 28 | 43 | K3 | Inferior parietal lobule (left) |
| 19 | 22 | 20 | 110 | -3.84 | 97 – 120 | 22 | 31 | K3 | Caudal orbitofrontal cortex (right) |
| -1 | -16 | 16 | 112 | 3.73 | 97 – 119 | 28 | 33 | K3 | Visual area V2 (left) |
| -23 | 6 | 26 | 120 | -3.55 | 79 – 120 | 25 | 40 | K3 | Inferior parietal lobule (left) |
| 13 | -8 | 32 | 111 | 2.99 | 103 – 116 | 25 | 28 | K3 | Inferior parietal lobule (right) |
| **MK2 (SMA: 45’ – 105’)** | | | | | | | | | |
| -23 | -2 | 24 | 80 | 4.99 | 45 – 105 | 57 | 97 | K5 | Caudal superior temporal cortex (left) |
| -15 | -10 | 32 | 96 | -4.25 | 45 – 105 | 53 | 140 | K4 | Visual area V4 (left) |
| 25 | 0 | 18 | 61 | 4.04 | 54 – 69 | 31 | 34 | K1 | Inferior temporal cortex (right) |
| 23 | -4 | 26 | 57 | -3.93 | 45 – 105 | 59 | 152 | K6 | Caudal superior temporal cortex (right) |
| -5 | 18 | 32 | 53 | -3.83 | 45 – 65 | 33 | 47 | K4 | Supplementary motor area (medial) |
| -1 | 28 | 28 | 84 | 3.82 | 72 – 105 | 34 | 48 | K3 | Anterior cingulate cortex (left) |
| 15 | 18 | 30 | 70 | -3.8 | 64 – 103 | 20 | 27 | K5 | Dorsal premotor cortex (right) |
| 23 | 14 | 10 | 62 | 3.77 | 56 – 71 | 21 | 22 | K1 | rostral superior temporal cortex (right) |
| -9 | 4 | 34 | 45 | -3.75 | 45 – 63 | 26 | 41 | K4 | Primary somatosensory cortex (left) |
| 15 | -18 | 26 | 84 | 3.65 | 54 – 96 | 24 | 32 | K2 | Visual area V1 (right) |
| 3 | -10 | 34 | 67 | -3.59 | 45 – 105 | 27 | 59 | K4 | Superior parietal lobule (right) |
| 19 | 8 | 26 | 51 | -3.51 | 45 – 68 | 32 | 55 | K4 | Inferior parietal lobule (right) |
| **MK3 (SMA: 65’ – 125’)** | | | | | | | | | |
| 15 | -2 | 32 | 89 | 5.47 | 65 – 125 | 946 | 1698 | K5 | Inferior parietal lobule (right) |
| -25 | 16 | 18 | 115 | -4.76 | 71 – 125 | 145 | 216 | K3 | Secondary somatosensory cortex (left) |
| -23 | -14 | 16 | 125 | -4.48 | 65 – 125 | 66 | 122 | K2 | Visual area V1 (left) |
| 25 | 22 | 16 | 116 | -3.77 | 65 – 125 | 120 | 260 | K3 | Ventral premotor cortex (right) |
| 27 | 8 | 18 | 89 | 3.76 | 65 – 110 | 47 | 67 | K5 | Caudal superior temporal cortex (right) |
| -5 | 18 | 28 | 80 | -3.27 | 76 – 94 | 20 | 23 | K1 | Midcingulate cortex (left) |
| 23 | -10 | 18 | 83 | -2.89 | 75 – 91 | 23 | 27 | K1 | Visual area V2 (right) |
| 21 | 24 | 20 | 83 | -2.78 | 76 – 91 | 22 | 24 | K1 | Ventral premotor cortex (right) |
| **MK1a (ACC: 80’ – 140’)** | | | | | | | | | |
| 9 | 10 | 4 | 88 | -6.69 | 80 – 130 | 20 | 29 | K4 | Parahippocampus cortex (right) |
| 19 | 14 | 18 | 132 | 5.47 | 80 – 140 | 44 | 61 | K5 | Insula (right) |
| 13 | 26 | 10 | 105 | 4.96 | 80 – 138 | 20 | 24 | K6 | Caudal orbitofrontal cortex (right) |
| 3 | 30 | 24 | 106 | -4.65 | 80 – 140 | 61 | 114 | K5 | Anterior cingulate cortex (right) |
| 7 | 2 | 10 | 112 | 4.48 | 86 – 140 | 24 | 35 | K5 | Visual area V3 (right) |
| -7 | 8 | 2 | 88 | 4.31 | 80 – 140 | 21 | 40 | K5 | Ventral pons (left) |
| 17 | 6 | 18 | 115 | -4.17 | 80 – 140 | 26 | 49 | K5 | Auditory cortex (right) |
| -17 | -4 | 28 | 112 | -3.82 | 80 – 140 | 25 | 60 | K5 | Inferior parietal lobule (left) |
| 1 | -22 | 26 | 108 | -3.71 | 82 – 140 | 54 | 132 | K5 | Visual area V1 (right) |
| -5 | 38 | 28 | 132 | -3.21 | 109 - 139 | 24 | 37 | K3 | Anterior cingulate cortex (left) |
| -5 | 14 | 30 | 80 | -2.89 | 80 – 92 | 20 | 31 | K4 | Midcingulate cingulate cortex (left) |
| **MK1b (ACC: 65’ – 125’)** | | | | | | | | | |
| 11 | 26 | 12 | 97 | 9.27 | 65 – 125 | 31 | 37 | K6 | Caudal orbitofrontal cortex (right) |
| 1 | 32 | 24 | 82 | -7.49 | 65 – 125 | 134 | 377 | K6 | Anterior cingulate cortex (right) |
| 5 | 10 | 2 | 72 | 6.88 | 65 – 125 | 43 | 61 | K6 | Ventral pons (right) |
| 21 | 18 | 16 | 109 | 6.77 | 65 – 125 | 24 | 24 | K6 | Insula (right) |
| -5 | 18 | 8 | 75 | -6.25 | 65 – 125 | 26 | 29 | K4 | Optic tract (left) |
| -19 | 22 | 12 | 98 | 6.23 | 65 – 125 | 41 | 56 | K6 | Insula (left) |
| -5 | 10 | 2 | 74 | 6.16 | 65 – 125 | 30 | 48 | K6 | Ventral pons (left) |
| -21 | 6 | 20 | 120 | -4.85 | 65 – 125 | 53 | 99 | K3 | Auditory cortex (left) |
| -1 | -4 | 0 | 72 | 4.73 | 54 – 102 | 21 | 30 | K4 | Dorsal medulla (left) |
| 1 | 0 | -8 | 107 | -4.71 | 65 – 125 | 26 | 39 | K6 | Ventral medulla (right) |
| 19 | 6 | 18 | 65 | -4.52 | 65 – 125 | 60 | 183 | K5 | Auditory cortex (right) |
| -1 | -6 | 18 | 124 | -4.09 | 92 – 125 | 49 | 78 | K2 | Posterior cingulate cortex (left) |
| 3 | 2 | 10 | 65 | -3.73 | 65 – 83 | 23 | 28 | K2 | Inferior colliculus complex (right) |
| 9 | -2 | 34 | 116 | -3.15 | 101 - 125 | 27 | 50 | K3 | Superior parietal lobule (right) |
| **MK2 (ACC: 50’ – 110’)** | | | | | | | | | |
| -1 | 36 | 24 | 50 | -24.15 | 50 – 110 | 140 | 252 | K6 | Anterior cingulate cortex (left) |
| -21 | 20 | 14 | 50 | 9.95 | 50 – 110 | 71 | 99 | K4 | Insula (left) |
| 21 | 14 | 18 | 50 | 7.57 | 50 – 110 | 57 | 105 | K4 | Secondary somatosensory cortex (right) |
| -9 | 6 | 10 | 85 | -7.32 | 50 – 110 | 178 | 314 | K6 | Hippocampus (left) |
| -9 | 22 | 8 | 110 | 6.94 | 50 – 110 | 39 | 53 | K6 | Amygdala (left) |
| 1 | 16 | 30 | 82 | 6.24 | 50 – 110 | 50 | 69 | K6 | Midcingulate cortex (right) |
| -13 | -10 | -2 | 107 | 4.29 | 50 – 110 | 21 | 34 | K6 | Intermediate cerebellar cortex (left) |
| 9 | -8 | 0 | 50 | -3.65 | 50 – 82 | 28 | 45 | K4 | Intermediate cerebellar cortex (right) |
| **MK3 (ACC: 40’ – 100’)** | | | | | | | | | |
| 7 | 20 | 8 | 100 | -9.29 | 40 – 100 | 109 | 190 | K6 | Amygdala (right) |
| -17 | 28 | 12 | 47 | -8.75 | 40 – 100 | 46 | 71 | K6 | Caudal orbitofrontal cortex (left) |
| 21 | 22 | 10 | 59 | -7.43 | 40 – 100 | 21 | 29 | K6 | Auditory cortex (right) |
| -23 | 8 | 24 | 84 | 5.32 | 40 – 100 | 23 | 29 | K6 | Inferior parietal cortex (left) |
| 1 | 6 | -2 | 48 | -5.12 | 40 – 100 | 26 | 56 | K4 | Ventral pons (right) |
| 1 | 36 | 22 | 48 | 5.09 | 40 – 100 | 47 | 95 | K6 | Anterior cingulate cortex (right) |
| 19 | 6 | 22 | 92 | 4.82 | 40 – 100 | 29 | 49 | K4 | Auditory cortex (right) |
| -1 | -20 | 14 | 70 | 3.12 | 70 – 78 | 21 | 38 | K4 | Visual area V1 (left) |
